# A Cross-Sectional Study of Work-Related Behaviour and Experience Patterns Among German Veterinarians in Different Age Groups

**DOI:** 10.3390/healthcare13192390

**Published:** 2025-09-23

**Authors:** Beatrice Thielmann, Emilia Döring, Robert Pohl, Irina Böckelmann

**Affiliations:** Institute of Occupational Medicine, Medical Faculty, Otto von Guericke University Magdeburg, Leipziger Str. 44, 39120 Magdeburg, Germany; robert.pohl@med.ovgu.de (R.P.); irina.boeckelmann@med.ovgu.de (I.B.)

**Keywords:** mental stress, job satisfaction, work behaviour, burnout, prevention, health promotion, occupational health prevention, occupational resilience, veterinary professionals, coping

## Abstract

Background/Objectives: The veterinary profession is associated with a variety of psychological stresses that increase the risk of exhaustion and burnout. There are no published systematic studies on work-related stress among veterinarians. The aim of this study was to apply work-related behaviour and experience patterns to this occupational group and to analyse age-related differences. Methods: A cross-sectional online survey was conducted among 832 practising veterinarians in Germany. The questionnaire was used to record work-related behaviour and experience patterns. The data were evaluated in terms of the frequency of the four AVEM patterns (G, S, A, and B) and age-related differences. Correlation and multivariate variance analyses were performed. Results: Overall, 61.1% of the respondents exhibited a risk pattern (A or B). Pattern B (burnout) was the most common, at 40.3%. Significant differences were found between age groups in several AVEM dimensions, particularly in terms of the tendency to resign in the face of failure, distancing ability, and experience of success at work (all *p* = 0.001), with older veterinarians showing more favourable values. The strongest correlation was weakly negative between age and work-related ambition (ρ = −0.262 with *p* < 0.001). Multivariate variance analysis of various AVEM dimensions and independent variables (e.g., gender, field of study, professional status, place of work, and age group) explained a maximum of 6.7% of the variance in the AVEM dimension of work-related ambition. Conclusions: This study fills an existing research gap by applying the AVEM model to the occupational group of veterinarians and identifying age-related differences in the experience of occupational stress. The high prevalence of patterns that are harmful to health underscores the importance of occupational health prevention and highlights the potential of the AVEM approach for occupational psychological assessment and intervention in veterinary practice.

## 1. Introduction

### 1.1. Mental Stress in the Veterinary Profession

Veterinary work is considered a systemically important, highly demanding profession that places considerable physical and psychological demands on practitioners [1,2,3]. In addition to medical responsibility and a diverse range of tasks, economic pressure, ethical conflicts, human–human interactions, and psycho-emotional situations involving animal suffering and death lead to considerable psychological stress [4,5,6,7]. This often leads veterinarians into a conflict between their love for their profession and animals and the expectations of animal owners [7,8,9,10]. In this context, many veterinarians experience constant emotional stress, which can lead to psychological exhaustion in the long term. Particularly relevant in this context is the phenomenon of compassion fatigue, a condition that results from repeated exposure to the suffering of other living beings [11,12]. In addition to physical exhaustion, this form of secondary traumatization manifests itself in emotional numbness, inner distancing, and a declining sense of professional meaning [12,13]. A study by Quedraogo et al. [12] showed that US veterinarians, especially those working in small animal medicine, exhibit symptoms such as secondary traumatization (58.9%) and compassion fatigue (35.5%) at above-average rates. The same study also revealed that 50.2% of veterinarians have high rates of burnout symptoms. Further, 50% of veterinarians in each of the three different employment categories experienced symptoms of burnout. In addition, veterinarians and veterinary students worldwide have been reported to have greater risks of depression and even suicide than other professional groups [14,15,16,17,18,19]. Male veterinarians have one of the highest suicide-related death rates among all professions considered, at 55.3 per 100,000 inhabitants (compared with 21.3 per 100,000 inhabitants for doctors, 21.2 per 100,000 inhabitants for dentists, 24.5 per 100,000 inhabitants for pharmacists, and 24.7 per 100,000 inhabitants for notaries), which is more than twice as high as that reported by the general population (24.9 per 100,000 inhabitants). The suicide rate for female veterinarians, at 19.3 per 100,000 inhabitants, is almost three times greater than the average rate for the female population worldwide (7.5 per 100,000 inhabitants) [16]. A study of 2208 US veterinarians showed that 41% had experienced severe psychological distress and 17.3% reported having suicidal thoughts within the previous 12 months [20]. A German study of 3118 veterinarians found that 27.8% of them suffered from depression, and 9.2% of the veterinarians had suicidal thoughts. Furthermore, as many as 32.11% of veterinarians were classified as being at an increased risk of suicide [21]. Another publication analysed the risk of burnout among 1053 practising veterinarians in three age groups (≤35 years, 36–45 years, and >45 years). The middle-aged group was the most emotionally exhausted, whereas younger veterinarians reported poorer performance. Overall, 50.9% of the participants reported some symptoms of burnout, with 14.6% of the total sample clearly at risk (≤35 years with 17.0%, 36–45 years with 15.8%, and >45 years with 11.0%) [22]. A Slovenian study involving 473 veterinarians found that 28.3% reported high levels of burnout. The study also revealed an imbalance between work and private life, with ethical conflicts and long working hours emerging as significant predictors of burnout symptoms. Notably, younger veterinarians and women were found to be disproportionately affected [23]. A Europe-wide study by Jansen (2024) involving over 14,000 veterinarians revealed that those at the beginning of their careers, as well as female veterinarians, are at the greatest risk of experiencing reduced mental well-being [24].

### 1.2. Theoretical Framework

Against the backdrop of these worrying findings, the key question is which individual and work-related factors influence the psychological stress experienced by veterinarians. It is also important to clarify how health-promoting or health-threatening behaviour patterns can be systematically recorded. From an occupational health perspective, this requires an instrument that is both scientifically sound and applicable in practice. In this context, the model of work-related behaviour and experience patterns (AVEM) has proven to be particularly suitable [25,26]. It allows for a differentiated analysis of work-related behaviour and occupational coping strategies in the face of psychosocial stress, and is also impressive due to its valid, time-saving, and economical application, for example, in the context of occupational health care or counselling sessions. As a structured questionnaire with a clearly defined typology, the AVEM enables the early identification of health-risk patterns of behaviour, such as overcommitment, resignation, or burnout tendencies. It thus provides valuable starting points for targeted preventive measures [25,26].

The AVEM is based on the premise that mental health in the workplace is not solely determined by external stress factors, but above all by how individuals deal with these factors. The procedure takes an approach that goes beyond purely symptom-based methods by recording personal attitudes, mindsets, resources, and subjectively experienced skills. It emphasizes the active involvement of the affected person in shaping their experience of stress through specific patterns of behaviour and experience, mental resilience, and emotional responses to occupational demands. The AVEM is based on a critical further development of the Type A behaviour concept proposed by Friedman and Rosenman [27]. They originally postulated a link between excessive commitment to performance and cardiovascular disease. However, current findings in health psychology make it clear that it is not commitment itself that is critical to health, but rather its combination with low psychological resilience, negative emotions, and a lack of recovery capacity or distance [28,29]. These insights are incorporated into the AVEM in the form of a resource-oriented approach based on the principles of salutogenesis according to Antonovsky [30]. The focus is not on the development of disease, but on the conditions that contribute to maintaining health. The aim is to identify potential behaviour patterns associated with health risks at an early stage and in a differentiated manner so that targeted interventions can be implemented before manifest illnesses occur. The AVEM is therefore suitable for behaviour-oriented measures, for example, in the context of counselling or coaching, as well as for structural changes in working conditions.

The specific composition of the AVEM dimensions, their classification into four patterns (G—health, S—protection, A—exertion, and B—burnout), and the methodological approach used in the survey are described in detail in the methodology section.

### 1.3. Relevance, Research Gap, and Aim of the Study

Building on this scientific framework, the question arises as to the extent to which health-related patterns differ between veterinarians of different age groups, particularly regarding potentially age-related stress or coping profiles. This forms the basis for the relevance and objective of the present study.

The mental health of veterinarians is increasingly becoming the focus of occupational health and health science research. Nevertheless, there is still a lack of differentiated findings on how employees in this profession from different age groups cope with the specific demands of their everyday work and which behavioural and experiential patterns are associated with health resilience or an increased risk of burnout. Although age or age groups have often been considered a potential protective or risk factor in many studies, it has rarely been the main focus of analysis. Previous studies on the psychological stress experienced by veterinarians are often limited in terms of methodology or content, for example with regard to differentiation by age group or consideration of job-specific stressors. Clearly presenting these research gaps would demonstrate why applying the AVEM model is particularly valuable in this study.

The aim of this study was to use the AVEM model to investigate the extent to which work-related behavioural and experiential patterns differ among veterinarians of different age groups. The focus was on health-promoting patterns such as pattern G (“health”) or pattern S (“protection”), as well as patterns that are potentially risky for health, such as pattern A (“effort”) and pattern B (“burnout”). Particular attention was given to the question of whether younger professionals in particular exhibit stressful patterns more frequently and whether a transition to health-promoting behaviours can be observed with increasing professional experience.

The findings will provide targeted impetus for preventive measures, such as age-differentiated training programs for young professionals or the design of health-promoting work structures throughout their career.

On the basis of previous empirical findings, the following hypotheses were formulated:

**Hypothesis** **1.** *Younger veterinarians show significantly more risk patterns of work-related behaviour in the AVEM (A and B) than older colleagues do.*

**Hypothesis** **2.** *The characteristics of the AVEM dimensions differ according to age group. With increasing age and professional experience, for example, distancing ability from work increases, and at the same time, the tendency to resign in the event of failure decreases.*

## 2. Materials and Methods

**Figure 1:**
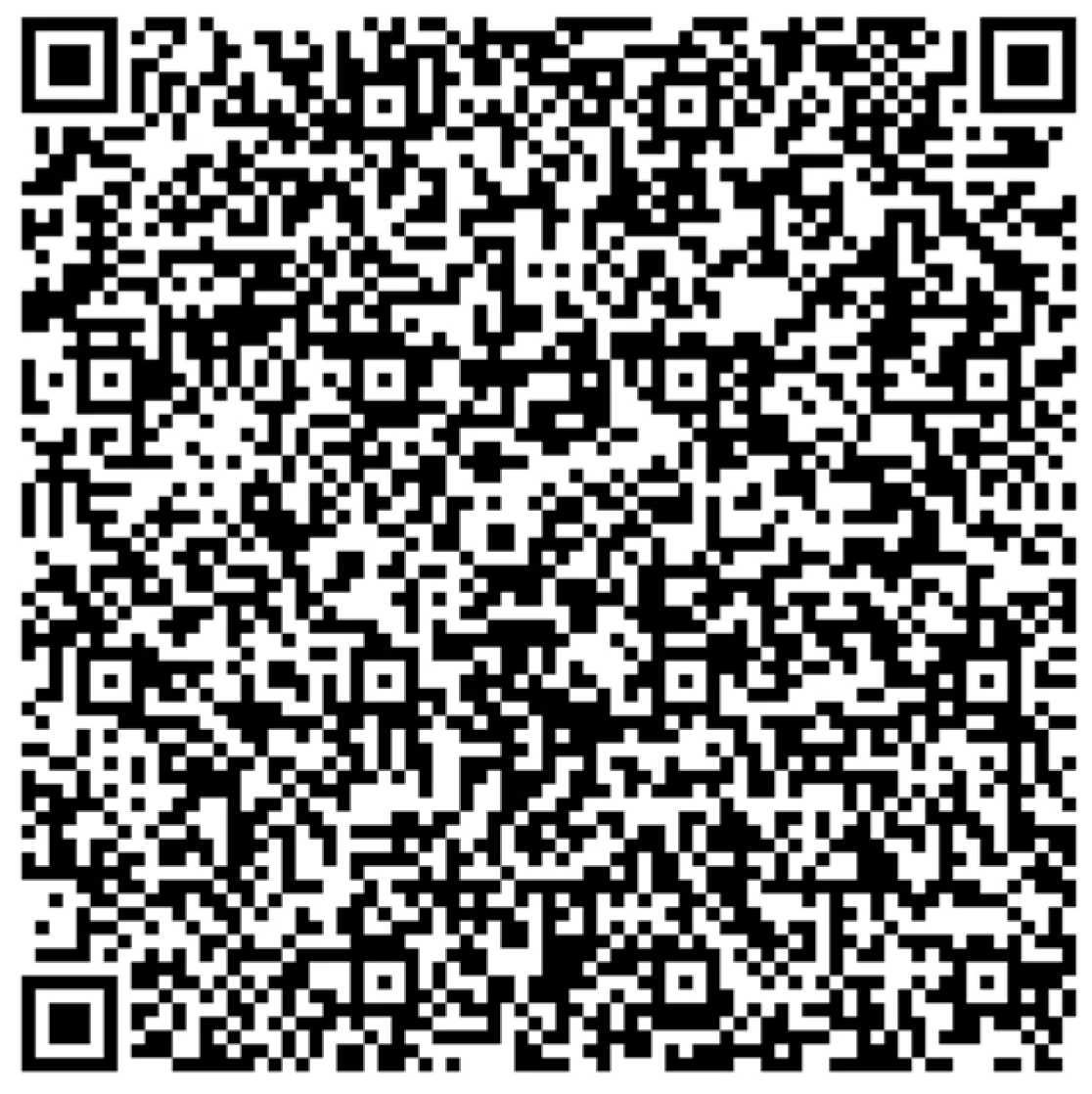
QR code for the study protocol.

The data presented here come from a cross-sectional study entitled “*Causes and consequences of psychological stress in the working life and emergency services of veterinary professionals in the Federal Republic of Germany*”. The research project was financially supported by the German Social Accident Insurance Institution for Health and Welfare Services (BGW) under grant number 1544. During the data-collection phase, there was cooperation with the Department of Veterinary Medicine of the State Office of Saxony-Anhalt. In addition, a positive ethics vote was obtained from the Ethics Commission of Otto von Guericke University Magdeburg (91/21). The study is officially listed in the German Register of Clinical Studies under registration number DRKS00026106. To recruit participants, the survey link was distributed via the state veterinary chambers and the German Veterinary Journal. Participation was anonymous and voluntary. The online survey was active from 1 July 2021 to 31 January 2022. The complete study protocol has been published [31] and is available online (see QR code Figure 1).

### 2.1. Sample

A total of 995 veterinarians aged between 23 and 79 years, recruited from across Germany, took part in the online survey. Due to incomplete questionnaires, 163 people were excluded from the final evaluation. Of the remaining 832 participants, 650 were female, corresponding to a proportion of 65.3%.

For the analysis, participants were divided into three percentile groups on the basis of their age. The variable “age group” (AG) comprised the following classification:

- First percentile ≤ 35.0 years (AG I);
- Second percentile > 35.0 to ≤45 years (AG II);
- Third percentile > 45.0 years (AG III).

Veterinarians in AG III were therefore considered older workers [32].

### 2.2. Methods

The study participants first provided sociodemographic data (age, gender, working years, marital status, and number of children in the household) and work-related factors such as employment status (self-employed, employed, and civil servant). In addition, the place of work (large city, medium-sized/small town, and rural area) and the field of work (large animals, small animals, and laboratory work) were taken into account.

A questionnaire on work-related behaviour and experience patterns (German: Arbeitsbezogenes Verhaltens—und Erlebensmuster, AVEM) according to [25,26,33] was used.

#### Work-Related Behaviour and Experience Patterns (AVEM)

The questionnaire on work-related behaviour and experience patterns (AVEM) is a versatile personality diagnostic tool for self-assessment of how individuals deal with professional demands [25,33]. It not only records symptoms of stress and strain, but also analyses fundamental attitudes and behaviours in the work context. The identified characteristics provide information about previous stressors and enable an assessment of personal resources for coping with future challenges. The focus is particularly on health-related behavioural and experiential patterns with the aim of identifying potentially risky strategies for mental health. Individual targeted measures can be derived on the basis of the results. The long version of the AVEM, with 66 items, covers 11 dimensions. The possible answers are on a 5-point scale: 5 = “completely true,” 4 = “mostly true,” 3 = “partly true/partly false,” 2 = “mostly false,” and 1 = “completely false.” The dimensions are assigned to three areas:

(a) Work engagement with the following dimensions: subjective importance of work, work-related ambition, willingness to work until exhausted, striving for perfection, and distancing ability;
(b) Resilience: again, distancing ability, tendency to resign in the face of failure, proactive problem solving, and inner calm and balance;
(c) Emotions: experience of success at work at work, satisfaction with life, and experience of social support [25,26]. The internal consistency of the standard version of the questionnaire is demonstrated by a Cronbach’s alpha value between 0.79 and 0.87, which indicates good to very good internal consistency of the procedure [26].

The evaluation begins with the addition of the raw values of the individual items to a total sum value. The standard values, known as the Stanine values, are then determined for the eleven dimensions. Based on a sample of 31,979 people, a 4-cluster solution was developed for assigning the behavioural patterns. These clusters represented the 4 patterns G, S, A, and B. Test subjects were assigned according to the predominant pattern [33] in accordance with the following classification into five pattern categories: complete, accentuated, tending, combined, or no pattern. Owing to the 4-cluster solution, only the 4 AVEM patterns were considered in this study. Combination patterns or study participants without a clear assignment were not included in the evaluation.

In the final step, the results were assigned to one of the four AVEM patterns, which are described in detail below [25,33].

- Pattern G—Health: Clear but not excessive characteristics can be identified in the area of work engagement. Work-related ambition is particularly pronounced, whereas the willingness to work until exhausted remains moderate. People with this pattern also have a pronounced ability to distance themselves from their work. In terms of resilience, there is a low tendency to resign in the face of failure, accompanied by pronounced proactive problem solving, inner calm, and balance. In addition, this pattern has the highest scores in the area of positive attitudes towards life. This includes, in particular, the dimensions of experience of success at work, satisfaction with life, and experience of social support.
- Pattern S—Protection: People who predominantly display pattern S are characterized by a pronounced tendency to resign in the face of failure. Striking features are low scores in the areas of subjective importance of work, work-related ambition, willingness to work until exhausted, and striving for perfection. Distancing ability from work is most pronounced in this group. However, low work engagement should not be confused with a resigned attitude, which is evident from the low tendency towards resignation. Overall, these individuals report a positive attitude towards life, which is characterized by high scores for inner calm and balance, satisfaction with life, and experience of social support. The tendency to protect oneself goes hand-in-hand with a fundamental satisfaction that is, however, predominantly anchored outside of professional life. More recent studies tend to argue that people with pattern S are not necessarily oriented towards self-protection, but have developed a protective mechanism against emotionally stressful work or a negative work environment [25,33]. Interventions of this type should therefore primarily aim to increase work motivation.

The following two risk types are characterized by work-related behaviour and experience patterns that endanger or impair mental health [33].

- Risk pattern A—Effort: People who expend excessive energy in their everyday working lives fall into this pattern. They attach great subjective importance to work, strive strongly for perfection, and invest considerable effort. At the same time, they show little distancing ability from their work. Their intense commitment makes them less resilient to stress. In addition, this type of person often lacks composure and has an increased tendency to resign in the face of failure. Negative emotions predominate, which is reflected in an overall lower level of satisfaction with life. Despite the high level of work-related ambition, this does not translate into positive emotions. Schaarschmidt and Fischer assigned risk pattern A to the concept of the gratification crisis described by Siegrist [33].
- Risk pattern B—Burnout: These individuals show many typical signs of burnout syndrome, as described by Maslach and Jackson, among others [34]. They have great difficulty separating themselves from their work, but their overall work-related ambition is low. They also exhibit a pronounced tendency to resign in the face of failure. This pattern is particularly characterized by the fact that affected individuals score lowest in the areas of proactive problem solving, inner calm, and balance. Their general well-being and satisfaction are rather low, which is reflected in weak scores in terms of perceived experience of success at work, experience of social support, and satisfaction with life.

### 2.3. Statistical Analysis

IBM SPSS Statistics Version 28 (Armonk, NY, USA) was used for statistical analysis. The Kolmogorov–Smirnov test was used to check for a normal distribution. After performing frequency analyses, percentile values for the age variable were calculated at 33% and 66% to form the three age groups.

Cross-tables were created, and Pearson’s chi-square tests were applied to examine the distributions of the age groups. In addition, Goodman and Kruskal’s tau tests were used to analyse the distributions of the individual subgroups in more detail. Descriptive statistics included the calculation of means, standard deviations, minimum and maximum values, and 95% confidence intervals of the means within the three defined age groups. The Kruskal–Wallis test was used to test for group differences, as the data were not normally distributed. The data were also tested using Bonferroni correction.

Only cases with complete data were included in the analysis; datasets with missing values for any of the variables were excluded.

In addition, nonparametric Spearman correlation analyses were performed, and the significance of the results was tested bilaterally. The correlation coefficients (ρ) were interpreted according to Cohen [35]: values from 0.10 to 0.29 were classified as weak, 0.30 to 0.49 as moderate, and ≥0.50 as strong.

To identify potential predictors for the AVEM patterns and dimensions, a general linear model (GLM) analysis with bootstrap specifications was performed, considering variables such as gender, type of employment, specialist area, and years of work experience. The effect sizes were classified according to Cohen [35]: η^2^ < 0.06 as a small effect, η^2^ between 0.06 and 0.14 as a moderate effect, and η^2^ > 0.14 as a strong effect.

## 3. Results

### 3.1. Sociodemographic and Professional Data of Veterinarians

In the present study, the demographic characteristics of 995 veterinarians were examined with respect to their age and professional experience (Table 1). The average age of the total sample was 41.7 ± 10.19 years. For further analysis, the samples were divided into three age groups (AGs). AG I comprised the youngest participants, with an average age of 31.0 ± 3.02 years. In AG II, the average age was 40.4 ± 3.02 years, and in AG III, it was 54.0 ± 5.49 years.

The participants in the overall sample had been working for an average of 14.2 ± 9.95 years. The participants in AG I had an average of 4.7 ± 2.80 years of working experience, those in AG II reported an average of 12.7 ± 4.79 years, and those in AG III reported an average of 25.6 ± 6.82 years. The differences between the age groups in terms of age and working years were highly significant (Kruskal–Wallis test: *p* < 0.001; Bonferroni correction: *p* < 0.001 in each case).

Table 2 shows the distributions of gender, marital status, and children in the household among the participants (*n* = 995), differentiated into three age groups (AG I: 23–35 years, AG II: 36–45 years, AG III: 46–79 years). Significant differences were found in all variables considered (*p* < 0.001).

The professional situation of veterinarians was analysed according to age group, and the results are presented in Table 3. Significant differences were found with respect to the type of employment, the field of specialization, and the type of contract (*p* < 0.001).

Employment: In AG I, the proportion of female trainees and assistant doctors was highest, at 38.9%, whereas in AG III, self-employment dominated at 67.3%. Employment in the public sector was relatively constant across all age groups (8.3–12.1%). Civil servant status was represented in only the two older groups. Employment in a practice or clinic was most common in AG I (28.0%), but decreased with age to 5.9% in AG III.

Specialization: In all age groups, small animals were by far the most common area of practice (between 52.8% and 57.1%). The use of large animal medicine decreased with increasing age (from 21.7% in AG I to 11.1% in AG III). The combined field of small animals, large animals, and laboratory work remained relatively constant, whereas work by public authorities was more common in the older groups.

Place of work: The differences between the age groups were not significant in terms of place of work (*p* = 0.352). A distinction was made between large cities, medium-sized and small towns, and rural areas.

Type of contract: Veterinarians in AG I were more likely to have fixed-term employment contracts (20.5%), whereas for those in AG III, the proportion of fixed-term contracts was very low, at 1.9%. The majority of the respondents had permanent contracts, especially in AG I (71.0%) and AG II.

### 3.2. Results and Mean Comparisons of the AVEM

**Figure 2:**
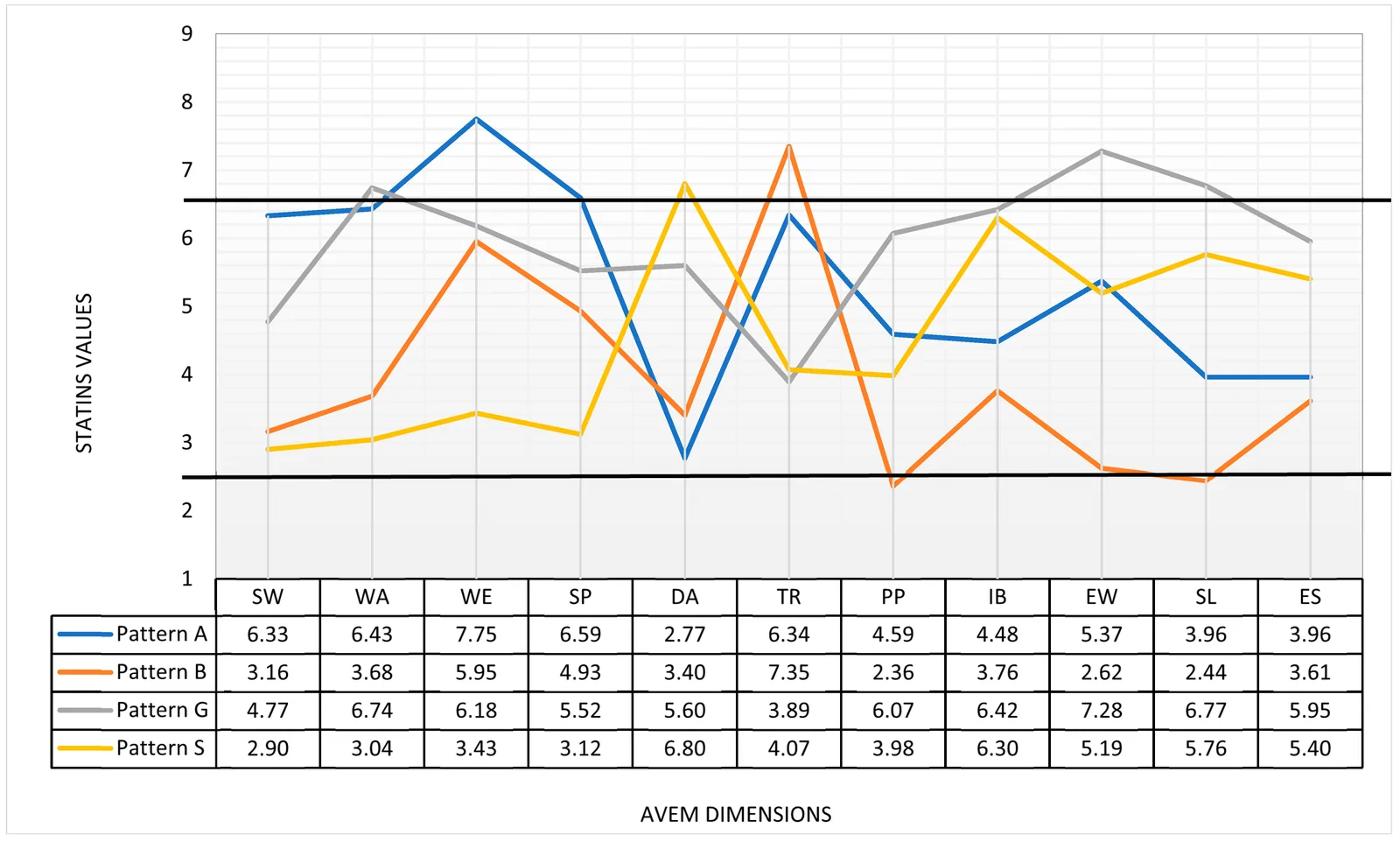
AVEM dimensions (Stanine) in a sample comparison (Pattern A, B, G, and S). Note: Normal range (3.5–6.5). SW = subjective importance of work, WA = work-related ambition, WE = willingness to work until exhausted, SP = striving for perfection, DA = distancing ability, TR = tendency to resignation in the face of failure, PP = proactive problem solving, IB = inner calm and balance, EW = experience of success at work at work, SL = satisfaction with life, ES = experience of social support.

Figure 2 provides an overview of the Stanine values of the AVEM dimensions, and Table 4 shows significant differences between the age groups.

The subjective importance of work was significantly greater among older veterinarians in AG III (4.4 ± 2.10) than among younger veterinarians in AG I (3.8 ± 1.94 with p_Bonferroni_ = 0.007) and middle-aged veterinarians in AG II (4.3 ± 1.90 with p_Bonferroni_; 0.001). Similarly, work-related ambition decreased with age, with younger veterinarians showing the highest values (5.4 ± 2.16 with p_Kruskal-Wallis_ *p* < 0.001). Distancing ability was most pronounced in the oldest group (4.7 ± 2.14), whereas younger veterinarians scored lower in this dimension (4.0 ± 2.09 with p_Bonferroni_) < 0.001). Significant differences were also found in the tendency to resignation in the face of failure. Older veterinarians were significantly less prone to resignation in the face of failure (5.4 ± 2.03) than younger veterinarians (6.3 ± 1.82 with p_Bonferroni_ < 0.001) and middle-aged veterinarians in AG II (5.9 ± 2.05 with p_Bonferroni_ = 0.002). The sense of professional success was also most pronounced in the oldest AG III (5.0 ± 2.28), whereas it was significantly lower among veterinarians in AG I (4.3 ± 2.22 with p_Bonferroni_ < 0.001) and veterinarians in AG II (4.5 ± 2.36 with p_Bonferroni_ = 0.008). Satisfaction with life was also highest in AG III (4.5 ± 1.96), with a particularly significant difference from that in AG II (4.0 ± 2.20 with p_Bonferroni_ = 0.008). In terms of experience with social support, younger veterinarians in AG I felt significantly more supported (4.9 ± 1.96 with p_Bonferroni_; 0.001 and 0.012, respectively) than their older colleagues. No significant age differences were found in the dimensions of willingness to work until exhausted, striving for perfection, proactive problem solving, and inner calm and balance.

A total of 724 participants could be assigned to one of the four AVEM patterns. Risk pattern B was the most common pattern, occurring in 292 individuals (40.3%), followed by risk pattern A, which was observed in 183 individuals (25.3%). Protective pattern S was observed in 152 veterinarians (21%), and health-promoting pattern G was the least common, with 97 cases (13.4%) (see Table 5). The analysis of pattern distribution in the different age groups did not reveal any significant differences, as the distributions across the groups were very similar (p_χ_^2^ = 0.181).

### 3.3. Correlation Analysis

Table 6 shows the results of a nonparametric correlation analysis according to Spearman’s rho (ρ) between age, years of professional experience, and various AVEM dimensions. Overall, increasing age and professional experience had both positive and negative correlations with AVEM characteristics. The most important results are summarized below. As expected, there was a very strong positive correlation between age and years of professional experience (ρ = 0.931; *p* < 0.001). The subjective importance of work was correlated with both age (ρ = 0.124; *p* < 0.001) and years of professional experience (ρ = 0.136; *p* < 0.001), i.e., it increased slightly with age. Work-related ambition, on the other hand, was negatively correlated with age (ρ = −0.262; *p* < 0.001) and years of employment in the profession (ρ = −0.223; *p* < 0.001). This indicated a decline in ambition with increasing age and working years. Distancing ability was positively correlated with age (ρ = 0.132; *p* < 0.001) and years of professional experience (ρ = 0.146; *p* < 0.001). This suggested an increasing distancing ability with age. The tendency to resign in the face of failure decreased with age (ρ = −0.170; *p* < 0.001) and professional experience (ρ = −0.154; *p* < 0.001). Further positive correlations were revealed between age and proactive problem solving (ρ = 0.064, *p* = 0.010); inner calm and balance (ρ = 0.065, *p* = 0.031); experience of success at work at work (ρ = 0.137, *p* < 0.001); and satisfaction with life (ρ = 0.064, *p* = 0.044). In terms of years of employment, there were parallel positive correlations with proactive problem solving (ρ = 0.113; *p* < 0.001); inner calm and balance (ρ = 0.105; *p* < 0.001); perceived success at work (ρ = 0.132; *p* < 0.001); and satisfaction with life (ρ = 0.105; *p* < 0.001). The experience of social support was negatively correlated with age (*p* = −0.097; *p* = 0.002).

### 3.4. Multivariate Analysis of Variance (MANOVA) of AVEM Stanines

Table 7 shows the results of a multivariate analysis of variance (MANOVA) on various AVEM dimensions and independent variables (e.g., gender, subject area, and age group) with regard to their effects. The majority of the independent variables significantly explained the variance in several AVEM dimensions. This is particularly true for the subjective importance of work (SB), BE, work-related ambition (BE), distancing ability (DF), tendency to resign in the face of failure (RT), experience of success at work (EE), and experience of social support (EU). However, there were no weak effect sizes (η^2^). The explained variance (corrected R^2^) remained low overall. At most, the model was able to explain 6.7% of the variance in the AVEM dimension of work-related ambition.

In terms of individual influencing factors, gender and occupational status had no significant influence on the AVEM dimensions examined. The field of study, on the other hand, showed significant correlations in several dimensions, particularly in the subjective importance of work (*p* = 0.007), willingness to exert oneself (*p* = 0.047), distancing ability, and tendency to resign in the face of failure (both *p* < 0.001). The effect sizes were only weak. The place of work also plays only a minor role. Only in the case of work-related ambition (*p* = 0.001) was there a significant difference, with a weak η^2^ value.

The age group appeared to be more relevant. It significantly influenced numerous AVEM dimensions, including the subjective importance of work, work-related ambition, distancing ability, tendency to resign in the face of failure, satisfaction with life, experience of success at work, and experience of social support. The effect sizes were weak.

## 4. Discussion

The aim of this study was to use the AVEM procedure to identify differences in the work-related behaviour and experiences of veterinarians in different age groups. The initial hypothesis was that younger veterinarians would exhibit risk patterns of work-related behaviour (patterns A and B) significantly more frequently than their older colleagues. However, this assumption could not be confirmed. Although risk pattern B occurred slightly more frequently in the youngest age group (AG I), at 41.4%, than in AG III (34.9%), and pattern A was also most common in AG I, at 27.8%, these differences were not statistically significant. On the basis of the available data, no age-related differences in the prevalence of risk patterns could be identified. However, it should be noted that veterinarians of all ages are constantly exposed to high levels of stress [1,3,7]. A comparison with other occupational groups is provided in Section 4.1. Possible explanations for this could lie in occupational stress factors that have a similar impact on mental health regardless of age. It is also conceivable that protective mechanisms and resources that older workers develop over time are not differentiated in the pattern distribution but rather in specific dimensions of the AVEM.

Hypothesis 2 was largely confirmed. Significant differences between the age groups were found in several AVEM dimensions. The distancing ability was significantly more pronounced among older veterinarians (AG III). This finding supports the assumption that the ability to emotionally distance oneself from occupational stress increases with increasing professional experience. The tendency to resignation in the face of failure decreased significantly with age, indicating greater frustration tolerance and psychological stability among older employees. Older veterinarians also scored significantly higher on measures of perceived success at work and satisfaction with life, suggesting a stronger professional identity and higher self-efficacy expectations. Moreover, work-related ambition was significantly more pronounced among younger veterinarians, which is plausible from an age psychology perspective and is consistent with life stage effects [36,37]. Interestingly, younger veterinarians reported significantly higher levels of social support, which could indicate stronger networking, possibly through digital communication channels, continuing education events, or collegial support in the initial phase.

These age-related differences were also reflected in the correlation analysis between the demographic variables of age and years of professional experience and the AVEM dimensions. Significant, albeit mostly weak, correlations were found between age and central AVEM dimensions. In particular, the negative correlation between age and the tendency to resign in the face of failure and the positive correlation with distancing ability confirm the postulated protective potential of professional experience. The results illustrate that the AVEM dimensions can serve as differentiated indicators of age-dependent stress and resource profiles, even if these are not reflected in the classic AVEM patterns.

In the multivariate analysis, the variable age group had significant but weak effects on seven of the eleven AVEM dimensions as follows: the subjective importance of work, work-related ambition, distancing ability, tendency to resign in the face of failure, experience of success at work, satisfaction with life, and experience of social support. This suggests that age alone does not have a significant influence on work-related behaviour or experience patterns. Rather, psychological stress in the veterinary profession appears to be independent of age and more strongly influenced by other factors.

### 4.1. Significance of AVEM Patterns for Occupational Health and Implications for Preventive Measures

The results of the present study are partly consistent with research findings from Germany and other countries on psychological stress and coping in the veterinary profession. Studies have repeatedly shown that veterinarians, similar to other health professionals, have an increased risk of work-related stress, burnout, and psychosomatic complaints [1,9,12,16,17,38]. Younger veterinarians are considered particularly at risk [22,39].

The pattern distribution in the veterinarians sample in this study revealed an increased occurrence of health-threatening AVEM patterns B (40.3%) and A (25.3%). The highest proportion of veterinarians with AVEM pattern A was found in AG I (27.8%), whereas AG II had the highest proportion of pattern B (44.6%). Employees and self-employed persons from 60 general practices with 84 doctors in management positions, 28 employed doctors, and 254 practice assistants presented only 8.4% AVEM pattern A and 19.5% AVEM pattern B [40]. Thus, the levels of AVEM patterns A and B among doctors and practice assistants were below those of the veterinarians surveyed in the present study [40]. Another cross-sectional study of 344 physicians also revealed a low prevalence of risk pattern A (12.6%) and risk pattern B (27.3%) [41].

The reported prevalence of AVEM risk patterns A and B varied significantly across different occupational groups. For example, while a particularly high proportion (65%) was reported among university lecturers [42], the figures for police officers (34%) and prison officers (38%) were significantly lower [43]. The prevalence among psychotherapy trainees was 47% [44], it was 41% among hospital nurses [45], and between 38% and 50% among geriatric nurses [46,47]. Medical students were particularly affected, with a rate of 69% [48], whereas employees of an international financial services company had a comparatively low rate of 34% [49].

A comparison of the experiences of veterinarians with those of other professional groups clearly reveals that targeted measures for prevention and health promotion are urgently needed. The results underscore the need for action at the individual level and the responsibility of organizations and institutions to create structural conditions that sustainably support the mental well-being of veterinarians. Therefore, possible intervention approaches and prevention strategies are presented and discussed in Section 4.4.

### 4.2. Theoretical Context

Compared with other well-established methods of diagnosing stress and strain, the AVEM approach has the advantage of not only recording symptoms and degrees of strain, but also health-related resources, as well as differentiated patterns of behaviour and experience. It thus goes beyond purely deficit-oriented methods. It should be acknowledged, however, that the eleven dimensions sometimes overlap in terms of content, which can lead to room for interpretation. Nevertheless, these “limitations” do not diminish AVEM’s usefulness as a practical screening instrument for prevention and counselling.

The results of this study can be interpreted well within the framework of the occupational psychology AVEM model. His model is based on the assumption that it is not primarily objective stressors but rather individual attitudes, resources, and subjective assessments of job requirements that are decisive for experiencing stress [25,26,33]. The observed differences in the AVEM dimensions between the age groups, for example, in their distancing ability, tendency to resignation in the face of failure, or the experience of success, reflect typical coping patterns that tend to stabilize with increasing professional experience. In particular, the increased distancing ability and the reduced tendency to resignation in the face of failure among older veterinarians indicate mature protective mechanisms in dealing with occupational stress [33]. The literature suggests that older employees are less likely to use passive-avoidant coping strategies than younger employees. Younger employees tend to engage in avoidance behaviour more often when they have little control over their work, whereas older employees in comparable situations tend to prefer more active, problem-solving strategies [50].

Furthermore, the findings can also be interpreted within the framework of Antonovsky’s salutogenesis theory [30], which advocates the basic belief that one’s own life is understandable, manageable, and meaningful. The higher scores among older veterinarians in the AVEM dimensions of experience of success at work, satisfaction with life, and inner calm and balance can be understood as an expression of a stronger sense of coherence. This enables individuals to use generalized resistance resources (such as experience, self-efficacy, and social support) to cope with stressful situations [30].

The theoretical positioning of the results provides a plausible framework for interpreting age-related differences in the AVEM dimensions and underscores the importance of individual resources in addressing occupational stress. Moreover, the results clearly indicate that effective stress management is not exclusively associated with age but is necessary in all age groups and can/must be specifically promoted. To emphasize the significance of the study, its central methodological strengths and limitations are explained in the following section.

### 4.3. Strengths and Weaknesses of the Study

By using a standardized, validated instrument such as AVEM, this study contributed to systematically classifying previous results from other occupational groups and making future studies methodologically compatible and more comparable. This study is notable for its large and heterogeneous sample, which represents different genders, employment groups, work locations, and specialist areas.

Nevertheless, his study also had limitations, such as the cross-sectional nature of the survey, which did not allow conclusions about causality. In addition, the survey was based on self-reported data; thus, distortion effects (e.g., social desirability) cannot be ruled out. Even though age-differentiated differences were found in several dimensions, the effect sizes remained small across the board, which points to the multifactorial nature of occupational stress. Selection bias due to voluntary participation, as participation occurred via veterinary chambers and online channels, may have led to an overrepresentation of particularly stressed or reflective individuals. Since not all participants could be clearly assigned to an AVEM pattern, the evaluation of the pattern distribution was based on a sample with slightly reduced size (*n* = 724). The inclusion of so-called “mixed types” was deliberately avoided, as these types are often characterized by overlapping characteristics in different dimensions that would reduce the clarity of the content. Mixing protective and risk factors within a pattern would significantly limit the validity of the results and make them more difficult to interpret. Therefore, mixed types were omitted in favour of a clearer type structure, even if this resulted in a reduction in the sample size for the pattern analysis.

The interpretation of the findings requires a degree of caution, given that age and professional experience are highly correlated and cannot therefore be evaluated independently. Differences between generations or disciplines may also influence experience and how professional stress is processed. Furthermore, while the AVEM approach focuses on individual patterns, structural and social factors such as working conditions, gender, and employment status may not be represented in sufficient detail in this study. Although gender-specific life course factors can influence work-related experiences, the potential for such effects has not yet been adequately demonstrated. Therefore, future studies should integrate life course variables and employment trajectories to better capture gender-specific stress patterns. This is particularly relevant given that most of the veterinarians surveyed in this study were female, and the proportion of women among veterinarians is generally increasing.

### 4.4. Practical Recommendations

A key starting point for early preventive measures is the study of veterinary medicine. The already high level of stress experienced by students should be taken into account, and prevention should be designed in such a way that it can be integrated into the curriculum without placing additional burden on students. Students should be made aware of mental health issues during their studies and be specifically prepared for professional challenges. It is recommended that practical content such as communication with animal owners, time management, and coping strategies be integrated into the curriculum [51,52]. Intervention studies have shown that practice-oriented communication training, for example, role-playing, is considered particularly effective by students [53]. One study examined the mental health, addictive behaviour, and internet use of veterinary students and teaching staff. A total of 226 people participated, including 177 students and 49 teachers. The results showed that students suffered more frequently from mental stress, greater substance use (e.g., alcohol and anxiolytics), and problematic internet use than teachers did. In particular, female students reported emotional stress and a greater need for psychological support. Younger age, excessive internet use, and the consumption of hookahs and anxiolytics were associated with lower satisfaction with life. The authors called for targeted measures to promote the mental well-being of veterinary students [54].

In addition to interindividual differences, such as professional experience or age, general recovery strategies also play a central role in coping with occupational stress. In their meta-analysis, Headrick et al. 2023 [29] showed that so-called “recovery experiences,” such as psychological detachment, relaxation, and experiencing control, were significantly associated with better mental health and greater work engagement [29]. These findings underscore the importance of targeted recovery promotion as a protective factor in everyday working life, including in the veterinary context.

Despite increasing awareness of this issue, there is still a lack of systematically evaluated interventions specifically tailored to veterinarians. Ethical aspects and dealing with ethically critical situations have also been inadequately addressed in university studies to date. A total of 78% of respondents in a Scottish study stated that they had not been adequately prepared for these ethical aspects [55]. According to the German Social Accident Prevention Institution for Health and Welfare Services (BGW), topics such as euthanasia and critical decisions are also considered psychologically stressful and should be included in the curriculum [56].

There are also important areas for action outside the university at a later stage. As a professional association, the German Association of Practicing Veterinarians (bpt) can help to shape political measures, for example, to limit working hours and promote family-friendly service models. Studies have shown that fewer night and weekend shifts are associated with greater job satisfaction [52,57]. However, gaps in care provision, especially in rural areas, must also be considered. Veterinary societies or veterinary associations also offer starting points for health-promoting measures and regular training.

Another key instrument is the workplace risk assessment required by law in Germany, which can be used as a starting point for concrete interventions [56]. The evaluation, alignment, adaptation, and (re)evaluation of all the measures are crucial to their success. In summary, considerable efforts are still needed to create jobs for veterinarians that are more supportive and attractive, and that promote well-being, a good work–life balance, and job satisfaction [24].

Furthermore, recommendations are made for dealing with AVEM risk patterns A and B within the framework of occupational health/company medical advice. For employees with AVEM risk patterns A and B, companies can target medical measures to promote mental health at the following points: measures for relaxation and physical balance, such as regular exercise, sports, breathing or mindfulness exercises, and regenerative leisure activities; programs or discussions that promote positive experiences in everyday working life and private life; realistically assessing professional goals and adjusting them if necessary, especially in the event of repeated failure or feelings of being overwhelmed; offering/accepting support in recognizing one’s own stress patterns; developing joint strategies for short-term and long-term stress management to increase individual relaxation; raising awareness of the importance of a supportive work environment with the establishment and maintenance of social contacts, including outside of work, and learning to say “no” appropriately; critically questioning work habits; structuring time and task management; addressing excessive work commitment to the point of exhaustion; providing training in tolerance to stimuli and frustration and in dealing with anger; and providing skills for solution-oriented and clear communication, especially in conflict situations. Coaching can be helpful when employees show signs of problems, withdrawal, hopelessness, or severe emotional stress, with a focus on self-awareness, self-confidence, emotion regulation, and anxiety management.

## 5. Conclusions

This study closed a research gap by systematically applying the established AVEM model to the veterinary profession for the first time, considering age-related differences in the experience of occupational stress. The results showed that age-related differences were particularly evident at the level of individual AVEM dimensions such as distancing ability, tendency to resign in the face of failure, or sense of achievement, whereas the distribution of risk patterns A and B was largely independent of age. This suggests that although professional experience leads to a more differentiated approach to stress, psychological strain affects all age groups.

The high prevalence of health-threatening AVEM patterns among veterinarians underscores the urgent need for targeted preventive measures at both the individual and organizational levels. The AVEM model has proven to be a valuable occupational health tool for the early detection of risk constellations and for deriving tailored intervention strategies in a professional context. Particularly in the context of occupational health care or counselling, it offers practical starting points for promoting mental health in the veterinary profession.

## Figures and Tables

**Table 1:** Age and working years in the different age groups.

Variable	Total Sample (*n* = 995)	AG I (*n* = 332; 33.4%)	AG II (*n* = 339; 34.1%)	AG III (*n* = 324; 32.6%)	p_Kruskal_ _Wallis_	p_Bonferroni_
	M± SD	MW± SD Median (min–max) [95% CI]				
Age (years)	41.72 ± 10.189	31.01 ± 3.023	40.42 ± 3.023	54.04 ± 5.486	<0.001	I–II < 0.001
		31 (23–35)	40 (36–45)	54 (46–79)		I–III < 0.001
		[30.69–31.34]	[40.13–40.72]	[53.44–54.64]		II–III < 0.001
Working years (years)	14.24 ± 9.948	4.70 ± 2.799	12.71 ± 4.791	25.62 ± 6.821	<0.001	I–II < 0.001
		5 (1–11)	13 (1–45)	25 (6–50)		I–III < 0.001
		[4.40–5.00]	[12.20–13.22]	[24.88–26.37]		II–III < 0.001

**Table 2:** Sociodemographic characteristics of participants by age group: gender, marital status, and presence of children in the household (*n* = 995).

Gender	AG I (*n* = 332)	AG II (*n* = 339)	AG III (*n* =324)	Total (*n* = 995)
	Number (% of Age Group)			Number
Male	96 (28.9%)	112 (33.0%)	137 (42.3%)	345 (34.7%)
female	236 (71.1%)	227 (67.0%)	187 (57.7%)	650 (65.3%)
Note: pχ^2^ according to Pearson ≤ 0.001; pGoodman-and-Kruskal–Tau < 0.001 (age group-dependent)				
**Marital**	**AG I** **(*n* = 332)**	**AG II** **(*n* = 339)**	**AG III** **(*n* = 324)**	**Total (*n* = 995)**
single	253 (76.2%)	197 (58.1%)	137 (42.3%)	587 (59%)
Married	78 (23.5%)	126 (37.2%)	147 (45.4%)	351 (35.3%)
Widowed	1 (0.3%)	2 (0.6%)	8 (2.5%)	11 (1.1%)
divorced	0 (0%)	14 (4.1%)	32 (9.9%)	46 (4.6%)
Note: pχ^2^ according to Pearson < 0.001; pGoodman-and-Kruskal–Tau < 0.001 (age group-dependent)				
**Children in the household**	**AG I** **(*n* = 250)**	**AG II** **(*n* = 245)**	**AG III** **(*n* = 251)**	**Total (*n* = 746)**
No	57 (22.8%)	119 (48.6%)	168 (66.9%)	344 (46.1%)
Yes	193 (77.2%)	126 (51.4%)	83 (33.1%)	402 (53.9%)
Note: pχ^2^ according to Pearson ≤ 0.001 pGoodman-and-Kruskal–Tau < 0.001 (age group-dependent)				

**Table 3:** Professional situation of veterinarians by age group: employment, specialty, place of work, and type of contract (*n* = 995).

Employment	AG I (*n* = 332)	AG II (*n* = 339)	AG III (*n* = 324)	Total (*n* = 995)
	Number (% of Age Group)			Number
Self-employed/practitioners	54 (16.3%)	129 (38.1%)	218 (67.3%)	401 (40.3%)
Employed in the public sector	30 (9.0%)	41 (12.1%)	27 (8.3%)	98 (9.8%)
Civil servants	0 (0%)	15 (4.4%)	17 (5.2%)	32 (3.2%)
Private sector/industry	13 (3.9%)	11 (3.2%)	6 (1.9%)	30 (3.0%)
Trainee lawyers/assistant doctors	129 (38.9%)	89 (3.2%)	34 (10.5%)	252 (25.3%)
Other	5 (1.5%)	8 (2.4%)	2 (0.6%)	15 (1.5%)
Not working	0 (0%)	0 (0%)	1 (0.3%)	1 (0.1%)
Doctoral candidates	8 (2.4%)	0 (0%)	0 (0%)	8 (0.8%)
Employed in practice/clinic	93 (28.0%)	46 (13.6%)	19 (5.9%)	158 (15.9%)
Note: pχ^2^ according to Pearson < 0.001; p_Goodman-and-Kruskal–Tau_ < 0.001 (age group dependent)				
**Subject**	**AG I** **(*n* = 332)**	**AG II** **(*n* = 339)**	**AG III** **(*n* = 324)**	**Total (*n* = 995)**
Small animals	184 (55.4%)	179 (52.8%)	185 (57.1%)	548 (55.1%)
Large animals (farm animals and horses)	72 (21.7%)	65 (19.2%)	36 (11.1%)	173 (17.4%)
Small animals and large animals	52 (21.7%)	44 (13.0%)	54 (16.7%)	150 (15.1%)
Laboratory	14 (4.2%)	11 (3.2%)	9 (2.8%)	34 (3.4%)
Government	10 (3.0%)	40 (11.8%)	40 (12.3%)	90 (9.0%)
Note: pχ^2^ according to Pearson < 0.001; p_Goodman-and-Kruskal–Tau_ < 0.001 (age group dependent)				
**Place of work**	**AG I** **(*n* = 332)**	**AG II** **(*n* = 339)**	**AG III** **(*n* = 324)**	**Total (*n* = 995)**
Large city (more than 100,000 inhabitants)	102 (30.7%)	80 (23.6%)	86 (26.5%)	268 (26.9%)
Medium-sized/small town (less than 100,000 inhabitants)	107 (32.2%)	119 (35.1%)	108 (33.3%)	334 (33.6%)
Rural areas	123 (37.0%)	140 (41.3%)	130 (40.1%)	393 (39.5%)
Note: pχ^2^ according to Pearson = 0.352; pGoodman-and-Kruskal–Tau = 0.347 (age group dependent)				

**Table 4:** Mean comparisons of the AVEM dimensions (stanine values) within the age groups.

Variable	Total Sample (*n* = 995)	AG I	AG II	AG III	p_Kruskal–_ _Wallis_	p_Bonferroni_
	M ± SD	M ± SD Median (min–max) [95% CI]				
Subjective importance of work	4.2 ± 2.00	3.8 ± 1.94	4.3 ± 1.90	4.4 ± 2.1,0	<0.001	I–II (0.007)
		4 (1–9)	4 (1–9)	5 (1–9)		I–III (<0.001)
		[3.59–4.01]	[4.07–4.47]	[4.21–4.67]		
Work-related ambition	4.8 ± 2.17	5.4 ± 2.16	4.8 ± 2.15	4.1 ± 2.01	<0.001	I–II (<0.001)
		6 (1–9)	4 (1–9)	4 (1–9)		
		[5.18–5.65]	[4.54–5.00]	[3.91–4.35]		I–III (<0.001)
						II–III (0.001)
Willingness to work until exhausted	5.9 ± 2.20	6.0 ± 2.22	6.1 ± 2.14	5.7 ± 2.24	ns	ns
		6 (1–9)	6 (1–9)	6 (1–9)		
		[5.71–6.19]	[5.88–6.34]	[5.46–5.95]		
Striving for perfection	5.0 ± 2.07	5.1 ± 2.05	5.1 ± 2.03	4.8 ± 2.12	ns	ns
		5 (1–9)	5 (1–9)	5 (1–9)		
		[4.87–5.32]	[4.89–5.32]	[4.57–5.04]		
Distancing ability	4.3 ± 2.11	4.0 ± 2.09 4 (1–9) [3.80–4.26]	4.2 ± 2.06 4 (1–9) [4.00–4.44]	4.7 ± 2.14 5 (1–9) [4.49–4.95]	<0	I–III (<0.001) II–III (0.008)
Tendency to resignation in the face of failure	5.9 ± 2.0	6.3 ± 1.82	5.9 ± 2.05	5.4 ± 2.03	<0.001	I–III (<0.001)
		7 (1–9)	6 (1–9)	6 (1–9)		
		[6.07–6.46]	[5.70–6.13]	[5.21–5.65]		II–III (0.002)
Proactive problem	3.86 ± 1.87	3.8 ± 1.78	3.8 ± 1.86	4.1 ± 2.0	ns	ns
solving		4 (1–9)	4 (1–9)	4 (1–9)		
		[3.56–3.94]	[3.57–3.97]	[3.84–4.27]		
Inner calm and balance	4.86 ± 1.88	4.8 ± 1.80	4.8 ± 1.89	5.0 ± 2.0	ns	ns
		5 (1–9)	5 (1–9)	5 (1–9)		
		[4.57–4.96]	[4.98–4.98]	[4.83–5.25]		
Experience of success at work	4.58 ± 2.30	4.0 ± 2.22	4.5 ± 2.3 6	5.0 ± 2.28	<0.001	I–III (<0.001)
		4 (1–9)	4 (1–9)	5 (1–9)		
		[4.06–4.53]	[4.21–4.71]	[4.75–5.25]		II–III (0.008)
Satisfaction with life	4.25 ± 2.25	4.2 ± 2.22	4.0 ± 2.20	4.5 ± 1.96	0.009	II–III (0.008)
		4 (1–9)	4 (1–9)	5 (1–9)		
		[4.68–5.11]	[4.01–4.42]	[4.29–4.80]		
Experience of social support	4.50 ± 2.01	4.9 ± 1.96	4.2 ± 1.95	4.4 ± 2.06	<0.001	I–II (<0.001
		5 (1–9)	4 (1–9)	5 (1–9)		
		[4.68–5.11]	[4.01–4.42]	[4.17–4.62]		I–III (0.012)

Note: ns = not significanFt.

**Table 5:** Distribution of AVEM patterns within the three age groups (number (%)).

AVEM Pattern	AG I (*n* = 237; 32.7%)	AG II (*n* = 249; 34.4%)	AG III (*n* = 238; 32.9%)	Total (*n* = 724)
	Number (% of Age Group)			Number
A	66 (27.8%)	59 (23.7%)	58 (24.4%)	183 (25.3%)
B	98 (41.4%)	111 (44.6%)	83 (34.9%)	292 (40.3%)
G	27 (11.4%)	34 (13.7%)	36 (15.1%)	97 (13.4%)
S	46 (19.4%)	45 (18.1%)	61 (25.6%)	152 (21%)

**Table 6:** Non-parametric correlation according to Spearman’s rho between the AVEM dimensions and age and years of employment.

AVEM Dimension	Age	Years in Occupation
Subjective importance of work	0.124 (<0.001)	0.136 (<0.001)
Work-related ambition	−0.262 (<0.001)	−0.223 (<0.001)
Willingness to work until exhausted	−0.038 (0.226)	−0.031 (0.332)
Striving for perfection	−0.045 (0.152)	−0.043 (0.179)
Distancing ability	0.132 (<0.001)	0.146 (<0.001)
Tendency to resignation in the face of failure	−0.170 (<0.001)	−0.194 (<0.001)
Proactive problem solving	0.064 (0.006)	0.113 (<0.001)
Inner calm and balance	0.065	0.105 (<0.001)
Experience of success at work	0.137 (<0.001)	0.200 (<0.001)
Satisfaction with life	0.064 (0.044)	0.105 (<0.001)
Experience of social support	−0.097 (0.002)	−0.054 (0.089)

Note. Correlation according to Spearman Rho 2-tailed (*p*-value). The more intense the colour, the stronger the correlation. Green = positive correlations, red = negative correlations.

**Table 7:** Generalized linear model or multivariate analysis of variance (MANOVA) of the AVEM stanines. Note: η2 = eta-squared, *p* = significance level. SW: subjective importance of work; WA: work-related ambition; WE: willingness to work until exhausted; SP: striving for perfection; DA: distancing ability; TR: tendency to resignation in the face of failure; PP: proactive problem solving; IB: inner calm and balance; SL: satisfaction with life; EW: experience of success at work; ES: experience of social support.

		SW	WA	WE	SP	DA	TR	PP	IB	EW	SL	ES
Corrected model	F	4.604	12.829	1.993	1.645	5603	9049	1789	1079	3878	2027	3526
	Corrected R^2^	0.021	0.067	0.006	0.004	0.027	0.046	0.005	0.000	0.017	0.006	0.015
	*p*	**<0.001**	**<0.001**	0.64	0.132	**<0.001**	**<0.001**	0.098	0.373	**<0.001**	0.060	**0.002**
	η2	0.027	0.072	0.012	0.010	0.033	0.052	0.011	0.007	0.023	0.012	0.021
Gender	*p*	0.888	0.293	0.697	0.636	0.696	0.015	0.528	0.710	0.242	0.592	0.709
	η2	<0.001	0.001	<0.001	<0.001	<0.001	0.006	<0.001	0.001	0.001	<0.001	<0.001
Subject area	*p*	**0.007**	0.719	**0.047**	0.080	**<0.001**	**<0.001**	0.152	0.864	0.085	0.382	0.947
	η2	0.007	<0.001	0.004	0.003	0.012	0.016	0.002	<0.001	0.003	<0.001	0.002
Occupational status	*p*	0.201	0.081	0.723	0.503	0.160	0.912	0.882	0.757	0.628	0.183	0.623
	η2	0.002	0.003	<0.001	<0.001	0.002	<0.001	<0.001	<0.001	<0.001	0.002	<0.001
Place of work	*p*	0.339	**0.001**	0.068	0.412	0.269	0.876	0.167	0.220	0.330	0.759	0.945
	η2	0.001	0.011	0.003	0.001	0.001	<0.001	0.002	0.002	0.001	<0.001	<0.001
Age group	*p*	**<0.001**	**<0.001**	0.068	0.117	**<0.001**	**<0.001**	0.090	0.119	**<0.001**	**0.012**	**<0.001**
	η2	0.020	0.057	0.005	0.004	0.017	0.024	0.005	0.04	0.015	0.009	0

## Data Availability

There are no plans to grant access to the full protocol, participant-level datasets, or statistical codes, as data contain potentially identifying information.

## Abbreviations

The following abbreviations are used in this manuscript:

AG	Age group
AVEM	German, Arbeitsbezogene Verhaltens- und Erlebensmuster; work-related behaviour and experience patterns
BGW	German, Berufsgenossenschaft für Gesundheitsdienst und Wohlfahrtspflege (BGW) Körperschaft des öffentlichen Rechts; Social Accident Insurance Institution for Health and Welfare Services
DA	Distancing ability
ES	Experience of social support
EW	Experience of success at work
IB	Inner calm and balance
PP	Proactive problem solving
SL	Satisfaction with life
SP	Striving for perfection
SW	Subjective importance of work
TR	Tendency to resignation in the face of failure
WA	Work-related ambition
WE	Willingness to work until exhausted
